# Machine Learning in Clinical Diagnosis of Head and Neck Cancer

**DOI:** 10.1111/coa.14220

**Published:** 2024-09-14

**Authors:** Hollie Black, David Young, Alexander Rogers, Jenny Montgomery

**Affiliations:** ^1^ Department of Naval Architecture, Ocean and Marine Engineering University of Strathclyde Glasgow UK; ^2^ Department of Mathematics and Statistics University of Strathclyde Glasgow UK; ^3^ Department of Otolaryngology, Head and Neck Surgery Queen Elizabeth University Hospital Glasgow UK

**Keywords:** area under the receiver operator curve, head and neck cancer, machine learning, up‐sampling

## Abstract

**Objective:**

Machine learning has been effective in other areas of medicine, this study aims to investigate this with regards to HNC and identify which algorithm works best to classify malignant patients.

**Design:**

An observational cohort study.

**Setting:**

Queen Elizabeth University Hospital.

**Participants:**

Patients who were referred via the USOC pathway between January 2019 and May 2021.

**Main Outcome Measures:**

Predicting the diagnosis of patients from three categories, benign, potential malignant and malignant, using demographics and symptoms data.

**Results:**

The classic statistical method of ordinal logistic regression worked best on the data, achieving an AUC of 0.6697 and balanced accuracy of 0.641. The demographic features describing recreational drug use history and living situation were the most important variables alongside the red flag symptom of a neck lump.

**Conclusion:**

Further studies should aim to collect larger samples of malignant and pre‐malignant patients to improve the class imbalance and increase the performance of the machine learning models.

**Summary**

- This observational cohort study's aim is to identify the machine learning model which best predicts head and neck cancer, through factors such as demographics, red flag symptoms or associated symptoms.
- After up‐sampling was conducted on the imbalanced dataset, the models evaluated were ordinal regression, lasso, elastic net, ridge, random forest, classification trees and linear discriminant analysis.
- Ranking was based on the multiclass area under the receiver operating characteristic (ROC) curve (AUC) and balanced accuracy (BA) and found that ordinal logistic regression (AUC = 0.6697, BA = 0.641) was the best performing model. With LDA (AUC = 0.6499, BA = 0.6047) and Ridge (AUC = 0.6529, BA = 0.6014) closely following.
- The three variables deemed to be most important were found to be the patient's living situation, drug use and having the symptom of a neck lump.
- Further studies should aim to collect more data on malignant and pre‐malignant cases or use different forms of up‐sampling to remove the class imbalance of the data presented in this study.

## 1 Introduction

Currently the number of patients referred to Urgent Suspicion of Cancer (USOC) diagnostic clinics are rising. Less than 10% of people referred to these clinics are diagnosed with cancer [1]. Within the Head and Neck clinic, malignant diagnosis pick‐up rates are even lower where the cancer pick‐up rate is between 3% and 8% [2, 3]. This high volume of patients attending for diagnoses has created a significant burden on the USOC head and neck referral pathway, making it challenging to meet the 31‐day diagnostic target created by the Scottish government.

The head and neck risk calculator has created a classification system, which can identify the probability of a patient having cancer based on their demographics and symptoms. The study obtained results with an AUC of 88.6% [4]. This study aims to review machine‐learning models and identify whether these algorithms can better predict head and neck diagnosis of cancer, to support USOC clinics.

## 2 Methodology

### 2.1 Data

There were 1045 patients eligible for inclusion in this observational cohort study. The reporting of this study adhered to the EQUATOR reporting guidelines for cohort studies. These patients were referred via the USOC pathway between January 2019 and May 2021. All patients included in the study agreed to anonymised data collection and analysis. Patients had to be adults over the age of 16 and had to have been referred to USOC. Return patients, patients with known active head and neck cancer and those who did not attempt the questionnaire were excluded from the study. Those who passed the inclusion criteria were categorised into three groups: patients with a benign diagnosis, diagnosis with malignant potential, and malignant diagnosis. There were 885 patients diagnosed with a benign condition, 61 patients with a diagnosis of malignant potential, and 99 malignant diagnoses.

For the purposes of subgroup analysis, benign, potential malignant and malignant diagnoses were sub classified and are shown in Table 1.

**TABLE 1:** Classification and frequency of diagnoses into benign, malignant potential and malignant outcome classification.

Benign Classification	Frequency	Malignant Potential Classification	Frequency	Malignant Classification	Frequency
No abnormality	301	Malignant potential salivary (pleomorphic adenoma)	29	Malignant oropharyngeal	26
Benign neck (sebaceous cyst, lipoma, thyroglossal duct cyst, branchial cyst, goitre, U2 nodules, reactive lymph nodes	298	Malignant potential thyroid (Thy3 follicular lesions)	18	Lymphoma	21
Benign pharynx (globus, benign oropharynx, reflux, benign oesophageal stricture)	146	Malignant potential laryngeal (laryngeal dysplasia)	11	Malignant laryngeal	23
Benign salivary (sialadenitis)	57	Malignant potential oral (leukoplakia)	3	Malignant thyroid—(Thy4 or 5)	13
Benign laryngeal (Reinke's oedema, presbyphonia)	54			Malignant hypopharyngeal	6
Musculoskeletal pathology (C‐spine pathology, sternoclavicular joint arthritis)	13			Metastatic SCC unknown primary	5
Benign oral	8			Synchronous H&N primaries	3
Granulomatous neck infection	7			Malignant salivary	1
Benign lateral skull base	1			Malignant oral	1
Total	885		61		99

### 2.2 Variables

The variables routinely recorded at clinic were a range of demographic questions, red flag symptoms and a questionnaire regarding other associated symptoms. Demographics included age, gender, employment status, living situation, smoking status, alcohol consumption and drug use. Red flag symptoms were persistent hoarseness, neck lump, persistent throat pain, an oral ulcer/lump, odynophagia or referred otalgia. Also included were associated symptoms reported throughout the questionnaire. These were cough, reflux, unexpected weight loss, dysphagia to solids and globus sensation.

### 2.3 Machine Learning

Due to the unbalanced nature of the data, the first machine learning approach taken was to up‐sample the data. Up‐sampling, also known as oversampling, is a method to modify the distribution of the data without having to decrease the size of the dataset and lose any important information. The method randomly duplicates rows of data from the class with low observations until the number of observations for this minority class is in line with the majority [5]. Prior to upsampling, the dataset underwent a repeated training and testing procedure, where the data was randomly partitioned into training and testing sets. This process was iterated 10 times, each with a unique split. The training set size varied between 65% and 90% of the total data, while the remaining portion was allocated for testing. Following each split, the training data underwent up‐sampling to address class imbalances.

The second machine learning approach taken was the modelling of the up‐sampled data. Seven models were created using four logistic regression‐based models (ordinal logistic regression, lasso, ridge and elastic net), two tree‐based models (random forest and classification trees) and lastly, a discriminant analysis model (linear discriminant analysis). The ordinal logistic regression based model was used as a comparison to conventional statistical techniques. Cross‐validation was also used within lasso, ridge and elastic net to obtain the optimal value of theta, a parameter within the model which represents the weighting given to the penalty term. Within all other models the parameters were kept to their default.

All analysis was conducted within R. To up‐sample the data the caret package was utilised. For the ML models the MASS, randomForest, rpart, and glmnet packages were used.

### 2.4 Model Comparison

To compare the models' predictive power, the multiclass area under the receiver operating characteristic (ROC) curve (AUC) was used. The pROC package was used to obtain the multi‐class AUC scores for each of the models, where the AUCs for each of the 10 iterations was averaged. Multiclass AUC is the mean of the one‐to‐one AUC scores [6]. A higher AUC score is considered better, with 1 representing perfect classification. If the score is 0.5 this means that the model predicts no better than a guess [7]. The macro average sensitivity and specificity was also calculated and averaged across the 10 iterations. The macro average is the same as how the multiclass AUC average is conducted, by deriving the mean of each of the one‐to‐one sensitivity and specificity values. The specificity identifies the proportion of true negatives that are correctly identified by the model and the sensitivity measures the proportion of true positives that are correctly identified. When this is averaged through macro averaging, it then indicates how the model performs among all classes. Due to the class imbalance of the data, the balanced accuracy was also selected for analysis of the models. For this, the “metrica” package in R was used and again the average of this was given for each of the 10 iterations of data split.

Each of the machine learning classification models chooses the most important variables, that have the most impact on the model. The logistic regression‐based and discriminant analysis models have model coefficients which explain the most impactful variables. The tree‐based models have a Gini impurity index which tells you the most important variables depending on their Gini score. These were also based on the 10 model iterations.

## 3 Results

### 3.1 Variable Descriptive Statistics

The demographics, red flag symptoms and associated symptoms in addition to descriptive statistics are outlined in Tables 2, 3, 4. From the demographics, a higher mean age is seen with malignancy and potential malignancy compared with benign. There are also more male patients who have malignancies, more retired patients, and a higher rate of patients with consumption of more than 14 units of alcohol per week. Potential malignancy had more smokers than the other categories. Throughout all participants, the rate of drug use is low, meaning the results that come from this variable should be treated cautiously.

**TABLE 2:** Demographics.

Characteristics Mean (SD); *n* (%)	Overall, *N* = 1045	1 Benign, *N* = 885	2 Malignant Potential, *N* = 61	3 Malignant, *N* = 99
Age (years)	52 (17)	51 (17)	56 (18)	61 (15)
Male	443 (42%)	351 (40%)	29 (48%)	63 (64%)
Employment status				
Employed	513 (50%)	451 (52%)	24 (41%)	38 (40%)
Full time education	42 (4.1%)	36 (4.1%)	4 (6.8%)	2 (2.1%)
No/Retired	475 (46%)	388 (44%)	31 (53%)	56 (58%)
Living Situation				
Married/living with partner/parents/children	746 (75%)	632 (75%)	44 (83%)	70 (74%)
Living with friends	18 (1.8%)	16 (1.9%)	0 (0%)	2 (2.1%)
Living alone	225 (23%)	194 (23%)	9 (17%)	22 (23%)
Residential care	2 (0.2%)	2 (0.2%)	0 (0%)	0 (0%)
Smoking status				
Never	468 (45%)	410 (47%)	23 (38%)	35 (36%)
Yes	255 (25%)	199 (23%)	26 (43%)	30 (31%)
Ex	308 (30%)	265 (30%)	12 (20%)	31 (32%)
Alcohol use				
Never	341 (35%)	295 (36%)	16 (28%)	30 (33%)
<14 units per week	510 (52%)	438 (53%)	31 (54%)	41 (45%)
>14 units per week	123 (13%)	93 (11%)	10 (18%)	20 (22%)
Drug use				
Never	939 (93%)	789 (92%)	58 (97%)	92 (98%)
Yes	31 (3.1%)	29 (3.4%)	2 (3.3%)	0 (0%)
Previously	44 (4.3%)	42 (4.9%)	0 (0%)	2 (2.1%)

*Note*: Age is a significant factor as most people are diagnosed after age 50. Cancer Research UK states that the highest rates in the UK are between the ages of 65 and 69.

**TABLE 3:** Red flag symptoms.

Characteristics	Overall, *N* = 1,045^a^	1 Benign, *N* = 885^a^	2 Malignant Potential, *N* = 61^a^	3 Malignant, *N* = 99^a^
Hoarseness	297 (31%)	243 (30%)	18 (33%)	36 (42%)
Neck lump	634 (64%)	525 (63%)	41 (72%)	68 (72%)
Throat pain	335 (35%)	289 (36%)	12 (23%)	34 (39%)
Oral Ulcer/Lump	193 (20%)	163 (20%)	6 (11%)	24 (27%)
It is painful for me to swallow food (odynophagia)	191 (19%)	147 (17%)	8 (14%)	36 (38%)
The pain travels to my ear (referred otalgia)	289 (29%)	240 (28%)	12 (21%)	37 (39%)

^a^ *n* (%).

**TABLE 4:** Associated symptoms.

Characteristics	Overall, *N* = 1,045^a^	1 Benign, *N* = 885^a^	2 Malignant Potential, *N* = 61^a^	3 Malignant, *N* = 99^a^
I cough a lot	213 (21%)	178 (21%)	10 (17%)	25 (27%)
I have heartburn or reflux	418 (41%)	366 (43%)	22 (39%)	30 (32%)
I have lost weight unexpectedly	141 (15%)	113 (14%)	9 (15%)	19 (20%)
I find it difficult to swallow solid foods like meats (dysphagia)	192 (19%)	154 (18%)	10 (17%)	28 (30%)
Feeling of something in throat	291 (33%)	243 (32%)	18 (35%)	30 (38%)

^a^ *n* (%).

Table 3 shows that malignant patients have experienced all six of the red flag symptoms more than benign patients. For potential malignant, these patients also experienced more occurrences of hoarseness and a neck lump as symptoms. However, fewer of them experienced throat pain, pain when swallowing, odynophagia and an oral ulcer/lump.

For the associated symptoms (see Table 4), cough, unexpected weight loss, dysphagia to solids and globus were experienced more in patients with malignancy. Less reflux symptoms were recorded in this group compared to benign diagnoses. Additionally, all these associated symptoms were experienced less or around the same overall as benign patients.

### 3.2 Machine Learning

The performances were ranked by multiclass AUC and balanced accuracy. The specificity and sensitivity were also both shown for a deeper understanding of the performance of the model, all results are shown in Table 5. Based on AUC and balanced accuracy, the ordinal regression model, the conventional statistical method, was the best performing model. This model also had the highest specificity and sensitivity. The second best model based on the AUC was Ridge, with LDA and the other two regularisation methods, lasso and elastic net closely following. Based on the balanced accuracy, LDA and classification trees are the top results, after the ordinal regression model. However, the balanced accuracy is similar throughout the models. High sensitivity is shown also in both classification trees and random forest, meaning that these models have the strongest performance in identifying true positives over the three classes. Although, the worst performing model's based on AUCs are the two tree based algorithms.

**TABLE 5:** Model performance.

Model	AUC	Balanced accuracy	Specificity	Sensitivity
Ordinal regression	0.6697	0.6410	0.7745	0.5076
Random forest	0.5874	0.6019	0.7385	0.4654
Classification tree	0.6261	0.6096	0.7470	0.4721
Lasso	0.6452	0.5967	0.7518	0.4416
Ridge	0.6529	0.6014	0.7553	0.4475
Elastic net	0.6435	0.5953	0.7521	0.4358
Linear discriminant analysis	0.6499	0.6047	0.7563	0.4564

The variable selection gave the most important variables for each model found in Table 6. The variables selected for lasso, ridge and elastic net were similar, selecting mainly the same variables for all three of the models. Although, ridge selected the variables ‘*Employment Status*’ and ‘*Gender*’ instead of ‘*Hoarseness*’ and ‘*Alcohol*’ which were selected by both lasso and elastic net. Both tree‐based algorithms choose the continuous variable (age) as the most important over any of the categorical variables; which contributes more data to the models than categorical variables, for these two algorithms. They also both chose ‘*Employment status’*, ‘*Alcohol’* and *‘smoking’* as their top variables. Ordinal logistic regression and linear discriminant analysis have chosen three of the same variables, these are also somewhat similar to those found for lasso, ridge and elastic net. The variable ‘*Drugs*’ is shown to be the top variable in 4 of the models, ‘*Living Situation’* and ‘*Neck Lump’* were also found to be associated in 4 and 5 of the models, respectively.

**TABLE 6:** Top five variables deemed to be important for each model.

Model	Important variables	Value
Ordinal logistic regression—variable coefficients (odds)	Neck lump	5.2572
	Alcohol	2.7730
	Hoarseness	2.5906
	Painful to swallow	2.2215
	Oral Ulcer/Lump	1.7932
Random forest—Gini impurity (Feature importance)	Age	312.9833
	Smoking	103.2749
	Alcohol	92.9403
	Employment	67.7636
	Living situation	60.9539
Classification trees—Feature importance	Age	189.3135
	Smoking	67.7430
	It is painful to swallow food (odynophagia)	46.7246
	Alcohol	43.5318
	Employment	42.1487
Lasso—Variable coefficients	Drugs	5.4829
	Living Situation	3.4077
	Neck lump	−1.4549
	Alcohol	−0.6572
	Hoarseness	−0.5846
Ridge—Variable coefficients	Drugs	2.7579
	Living situation	2.5753
	Neck lump	−1.0346
	Employment	−0.7188
	Gender	0.5159
Elastic Net—Variable coefficients	Drugs	5.0142
	Living situation	3.3717
	Neck lump	−1.4328
	Hoarseness	−0.6788
	Alcohol	−0.6565
Linear discriminant analysis—Variable coefficient	Drugs	−2.1931
	Neck lump	1.3147
	Gender	−0.9436
	Alcohol	0.8497
	Hoarseness	0.7373

## 4 Discussion

A number of variables were consistently selected for by multiple machine learning models as being predictive of risk for an underlying malignant diagnosis. This included a patient's socioeconomic status encompassing *‘living situation’*, *‘employment status’*, *age*, *‘drug use’*, ‘*alcohol*’, ‘*gender*, *‘smoking*’ and the red‐flag symptoms of ‘*neck lump’*, *‘odynophagia*’, and ‘*hoarseness*'. We will discuss the limitations of our results and how we mitigated for the unbalanced dataset, which was assembled.

### 4.1 Living Situation and Employment Status

A patients living situation can significantly impact their health outcomes, influenced by social, economic, and environmental factors. Our analysis found that residing in a care facility is associated with benign diagnosis. Despite common comorbidities and older age being a confounding risk factor for HNC [8], this finding is surprising. However, given the small percentage of patients within this category, the low numbers may have strongly influenced the ML models.

Although not evident in our data, other living situations may also impact the risk of developing HNC. For instance, living alone could signify social isolation and loneliness. Previous research links being male, single, and ‘never married’ with HNC risk [9]. Some studies also suggest that the stress of social isolation could contribute to head and neck carcinogenesis and tumour growth [10].

Our results have also identified ‘*employment status’* as a key variable in predicting malignant diagnosis, with being employed associated with benign diagnosis. The role of age may confound the association between HNC diagnosis and *‘unemployed/retired’* employment status, as age is an independent risk factor for HNC [11]. However, the association between unemployment and HNC is well‐documented, and has previously been investigated in the West of Scotland [12, 13, 14].

### 4.2 Drug Use

This study's findings reveal higher drug use prevalence in the benign group, opposing previous research. Woodley et al. suggested that drug use be evaluated as a red flag for those with suspected laryngeal cancer due to its association with increased burden of disease [15]. Similarly, Douglas et al. found that drug use is a risk factor for patients with laryngeal cancer, with the study finding a large odds ratio of association with disease [16]. We should however, note that patients often underreport such variables, impacting results.

Age may confound the perceived protective effect of drug use, as its more common in younger populations. Due to low participant drug use, its significance may be misinterpreted, considering the peak age of HNC is between 70 and 74 years [11].

### 4.3 Characterising the Significance of Neck Lump, Odynophagia and Hoarseness

Another significant variable in the study was the presence of a neck lump, often a key symptom of HNC [4]. However, our study found the second most common diagnosis among participants to be benign neck lumps, including reactive lymph nodes, sebaceous cysts and benign thyroid nodules. Malignant lumps exhibit different behaviours and presentation compared to benign ones as they typically enlarge progressively, rather than fluctuating in size like benign ones.

Similarly, odynophagia, or *‘painful swallow’* is a well‐recognised red flag symptom for HNC [4]. However, clinical suspicion for underlying neoplastic process can be guided by certain characteristics of the painful swallow. Odynophagia that is constant, lateralising, and travels to the ear is more concerning for a malignant process than odynophagia that is intermittent and felt in the midline. There are many alternative causes for persistent throat discomfort, including throat clearing, chronic cough, laryngopharyngeal reflux, and inhaler use.

Previous studies have established a significant association between hoarseness and HNC. The HaNC‐RC v2 [4] study confirmed that persistent hoarseness is significantly linked to an HNC diagnosis. Likewise, another study reported an odds ratio of 4.97 for individuals experiencing persistent hoarseness [16].

### 4.4 Smoking and Alcohol

This study also finds associations between smoking, alcohol and HNC diagnosis. Prior research confirms this with findings that current or ex‐smokers having a higher risk of HNC than non‐smokers. Similarly, alcohol consumption, especially exceeding 14 units per week, is associated with HNC [4].

The population‐attributed risk for head and neck cancer based on the consumption of tobacco and alcohol was also examined previously. The total risk of the consumption of tobacco and alcohol was found to be 72% [8].

### 4.5 Limitations and Future Work

The main challenge in modelling this data was its imbalanced nature. Out of the 1045 patients, 885 were diagnosed as benign, consistent with the 3%–8% malignant diagnosis rate in HNC clinics [3]. An unbalanced dataset results in high pick‐up rate for benign diagnosis, making It statistically effective but clinically unhelpful. As such, comparing a comparatively modest cohort of malignant diagnoses limits the ability to develop machine learning diagnostic tool with both high sensitivity and specificity.

Upsampling countered this issue, enhancing model performance. The ordinal logistic regression model showed the best performance, with LDA and regularisation techniques close behind. However, tree‐based algorithms underperformed, possibly due to overfitting from upsampling. Future work could explore alternative upsampling methods like Synthetic Majority Oversampling Technique (SMOTE) and consider deep learning techniques like Neural Networks (NN). Though a previous study favoured ML models over NN in palatal surgery outcome prediction [17], NN may excel in HNC diagnosis due to more predictor variables and a higher number of patients.

### 4.6 Future Clinical Use

As previously outlined, the Head & Neck Risk Calculator (HaNC‐RC v2) [4] is a screening tool for head and neck cancer, aiding GPs in determining the need for USOC referrals and minimising delays. The calculator was successfully used in secondary care during the COVID‐19 pandemic, primarily through telephone triage. However, with the return of face‐to‐face work post pandemic, this resource is no longer available. This present study was conducted after the patient was seen in outpatients and the risk calculator was not applied post hoc.

While identifying referrals that are high risk for cancer is an important feature of this machine‐learning diagnostic tool, our vision is that this diagnostic tool will also be able to reliably diagnose non‐malignant referrals, which make up the vast majority of the patients seen on the USOC pathway. In our application of this tool, in theory the GP has already used the risk calculator and have made the USOC referral. Then the patients are asked to complete our questionnaire and the referrals undergo re‐stratification. The risk calculator and our machine learning diagnostic tool are intended to work in tandem and are not necessarily mutually exclusive.

Our questionnaire encompasses almost all data points gathered by the HaNC‐RC V2, along with supplementary ones. Notably, predictors such as nose breathing and persistent head and neck skin lesions, which were identified as significant, were not incorporated in this study. Their inclusion could potentially enhance the multi‐class AUC of the ML models. Additionally, HaNC‐ RC v2 had a much larger population size for their study of 3500 contributing to their higher overall AUC. This is a limitation we would seek to build on in future work.

## Author Contributions

H.B. was involved in the analysis and interpretation of the data and manuscript preparation, D.Y. was involved in the conception and design of the study, A.R. and J.M. were involved in the acquisition of the data, and A.R., J.M. and D.Y. were involved in the manuscript editing.

## Ethics Statement

The authors have nothing to report. Study aims were designed to be in the public interest, with satisfactory methodological quality. High standards of confidentiality and data security were maintained throughout the study. Accordingly, the study was deemed to be low risk by the UK Statistics Authority, Ethics Self‐Assessment Tool [18].

## Conflicts of Interest

The authors declare no conflicts of interest.

### Peer Review

The peer review history for this article is available at https://www.webofscience.com/api/gateway/wos/peer‐review/10.1111/coa.14220.

## Data Availability

The data that support the findings of this study are available from the corresponding author upon reasonable request.
